# Short-term use of resveratrol in alloplastic graft material applied with calvarial bone defects in rats^1^

**DOI:** 10.1590/s0102-865020190070000004

**Published:** 2019-09-12

**Authors:** Nihat Laçin, Engin Deveci

**Affiliations:** I PhD, Assistant Professor, Department of Oral and Maxillofacial Surgery, Faculty of Dentistry, University of Katip Çelebi, İzmir, Turkey. Technical procedures, manuscript preparation and writing, final approval.; II PhD, Professor, Department of Histology and Embryology, Faculty of Medicine, Dicle University, Diyarbakir, Turkey. Technical procedures, histopathological examinations, manuscript preparation and writing, final approval.

**Keywords:** Skull, Resveratrol, Osteopontin, Osteonectin, Rats

## Abstract

**Purpose:**

The effects of resveratrol administration on calvarial bone defects with alloplastic graft material was investigated for osteoinductive reaction and bone development in rats.

**Methods:**

Healthy male rats were randomly divided into 3 groups consisting of 10 rats. Groups were as follows: control (defect) group, defect + graft group, and defect + graft + resveratrol group. A calvarial bone defect was created in all groups, alloplastic bone grafts were applied to the defect in the 2nd and 3rd group, resveratrol (5 mg/kg/day) was added to the drinking water of the animals following graft application for 28 days in the 3rd group.

**Results:**

Increase in osteoclasts and necrotic changes were observed histopathologically in the control group. In the 2nd group, reduction of inflammation, congestion of blood vessels, increased osteblastic activity, osteoinductive effect, progression of osteocyte development and increased collagen fibers in connective tissue were observed. In the 3rd group, osteoblasts seemed to secrete bone matrix and accelerate osteoinductive effect with increased osteopregenitor activity and positive osteopontin and osteonectin expressions.

**Conclusion:**

Resveratrol treatment was thought to be an alternative and supportive drug for implant application by inducing new bone formation in the calvaral defect region as a result of short-term treatment.

## Introduction

Bone defect, which occurs due to various reasons (such as trauma, infection) leads to serious problems^1^. Bone defects can be treated and reconstructed by various surgical methods, for instance, application of different graft models; e.g. cortical grafts, provide a durable and rigid structure but they have no ability to increase osteogenesis and, as they lack of vascularization, necrotic bone cannot repair itself. Other options are cancellous bone and bone marrow; the primary advantage of these is that they are able to enhance osteogenesis significantly^2^. Meanwhile, alloplastic bone grafts are synthetic, inorganic, biocompatible, and bioactive bone substitutions which are believed to repair bone defects through osteoconduction. Allografts are used freeze-dried demineralized bone allograft (FDBA) and demineralized freze dried bone allograft form known as DFDBA^3^. Wang *et al.* ^4^ suggested that lactic-coglycolic acid- alendronate may be a potential bone graft substitute to enhance bone repair in a rat femoral bone defect model. It was also reported that alendronate enhanced the new bone formation by autogenous bone graft in the rat calvarial defect model^5^. Experimentally induced calvarial critical dimension defects have been widely used to evaluate bone regenerative materials. In such a defect, it is important to maintain a suitable area due to the competition between the surrounding soft tissues and bone formation into the defect by using barrier membranes^2^. For the rat calvarial defect, 8 mm is generally accepted to be of critical size^6^.

Resveratrol (trans-3,40, 5-trihydroxystilbene, RSV) is a naturally occurring polyphenol and stilbene derivative, obtained from food sources such as red wine and numerous fruits including grapes, peanuts, nuts, pistachios, cocoa, berries and some Asian medicinal herbs^7^ ^,^ ^8^. A review by Murgia *et al.* ^9^ stated that RSV is a natural compound proven to possess both anti-inflammatory and anti-oxidant properties as a free radical scavenger even at low doses, regulates lipid metabolism, inhibits platelet activation and aggregation and also has bone protective properties. RSV has been studied for its several biologic effects and potential as a disease preventing agent in prevention and treatment of a wide variety of chronic diseases such as diabetes, cancer and dental caries. An experimentally induced RSV administered periodontitis model by Ikeda *et al.* ^10^ revealed healing of bone related to decreases in local oxidative damage and osteoclast activity. Boissy *et al.* ^11^ showed that RSV dose dependently stimulates the mRNA expression of the two osteoblastic markers osteocalcin and osteopontin in the immortalized osteoblast-like cells hMSC-TERT. According to Ornsturp *et al.* ^12^, RSV seems to inhibit proliferation and increase differentiation of osteoblasts independently of the inflammatory state.

Osteonectin is a glycoprotein synthesized by cells of an osteoblastic lineage that is abundantly expressed in bones undergoing active remodeling. Osteonectin is expressed by osteoblasts and odontoblasts^13^ and plays a role in the mineralization of bone and cartilage matrices^14^, cell-matrix interactions, and collagen binding. Osteonectin also increases the production and activity of matrix metalloproteinases so used as an indicator of new bone formation^15^. Osteopontin (OPN) secreted by the osteoblasts, is one of the major non-collagenous proteins. Studies concerning the expression of mRNA show a low expression of OPN during the maturation of the rat calvarium and the tibia in early bone formation^16^. OPN is produced by osteoclasts as well as differentiated osteoblasts and osteocytes and plays a role in resorption along with the formation, migration, and attachment of osteoclasts^17^. Baloglu *et al.* ^18^ reported that they induced bone matrix development in rats with clomiphene citrate after ovarectomy and induced new bone formation with increased osteonectin and osteopontin expression.

There is a gap in the literature on the basis of immunohistochemical studies about the use of resveratrol in calvarial bone defects with alloplastic graft material. So here, we aimed to investigate and provide an increase in the understanding of the effects of low-dose resveratrol administration on calvarial bone defects with alloplastic graft material application whether it acts in osteoinductive reaction and bone development.

## Methods

### Experimental procedure and groups

The investigation was conducted in accordance with the Guide for the Care and Use of Laboratory Animals published by US National Institutes of Health (NIH Publication no. 85-23, revised 1336). All procedures performed in this experiment were approved by the Ethics Committee for the Treatment of Experimental Animals (Faculty of Medicine, University of Dicle, Turkey). The rats (280-310g) were maintained under 23±2°C and 12 h light/dark cycles with *ad libitum* access to standard pelleted food and water. Animals were fed with standard laboratory food and water. All rats till the end of the analysis were healthy and no distinction in nourishment/water consumption and body weight pick up amongst experimental and control rats were noticed.

Three groups (10 rats per group) were arranged as below:

1. **Control (defect) group:** 8 mm calvarial bone defect was sutured without any treatment. The subjects were sacrificed at the end of the 4^th^ week.
2. **Defect + graft group:** 8 mm calvarial bone defects were created in all rats and then alloplastic bone grafts were applied to the defect. The subjects were sacrificed at the end of the 4^th^ week.
3. **Defect + graft + resveratrol group:** Alloplastic bone graft was placed in the calvarial bone defect and then, resveratrol (5 mg/kg/day) was added to the drinking water of the animals following the graft procedure for 28 days. They were sacrificed at the end of the 4^th^ week.

### Calvarial defect procedure

The animals were anesthetized with intraperitoneally 3 mg/kg xylazine (Rompun® 2%, Bayer Kimya San. Ltd. Sti., Istanbul, Turkey) and 90 mg/kg Ketamine HCl (Ketalar®, EWL Eczacibasi Warner Lambert Ilaç Sanayi ve Ticaret A.S., Istanbul, Turkey)^19^. Skin was incised to open frontal bone. A periosteal flap was removed with a thin elevator. Surgical sites were exposed with an incision through the skin and the periosteum at the midline of the calvaria. The periosteal flap was removed with a thin periosteal elevator and a specially designed trephine bur was created with a circular full-thickness bone defect with a diameter of 8 mm on the midline.

### Graft application

The Allograft material placed in the defect area of Group 2 and 3 was Biograft® HT (IFGL Bio Ceramics) which contains 40% β-Tri Calcium Phosphate with 60% porous biphasic synthetic Hydroxyapatite. This material is an alloplast with granule size of 350-500 μm with osteoconductive properties. Subcutaneous tissue was sealed with 6/0 vicryl suture and skin was allowed to heal.

### Resveratrol administration

Resveratrol was obtained from SIGMA Chemical, (Pool, Dorset), it was added to the drinking water of the animals following the graft procedure, and was given at 5 mg/kg/day for 4 weeks^20^. The drinking solutions were changed twice per week and protected from light in animal drinking bottles. Each rat belonging to the defect + graft + RSV group was housed individually in cages and had a volume of RSV solution in relation to body weight. Body weight was recorded weekly throughout this group and the animals in each group were monitored daily for general health and we made sure they were consuming the drinking water.

### Histologic examinations

At the end of the study, animals were anesthetized with intraperitoneally 3 mg/kg xylazine and 90 mg/ kg Ketamine HCl; then all animals were sacrified by decapitation. The skin, as well as all of the soft tissues surrounding the calvarial bone were removed. The samples were fixed with 10% neutral buffered formalin solution and decalcified with 5% EDTA (Ethylene dimine tetra acetic acid). After rinsing with tap water, the samples were dehydrated in increasing concentrations of ethanol and embedded in paraffin. Tissue sections of 4-6 μm thickness (RM2265 rotary microtome; Leica, Germany) were prepared in the transverse plane and stained using Hematoxylin-Eosin (H-E) staining for light microscopy examination.

Hematoxylin - Eosin staining procedure was as follows;

After the deparaffinizeing procedure of sections with 2 changes of xylene for 10 minutes each, they were re-hydrated in 2 changes of absolute alcohol, 5 minutes each. After being applied with 95% alcohol for 2 minutes and 70% alcohol for 2 minutes, sections were washed briefly in distilled water. Then, sections were stained in Harris hematoxylin solution for 8 minutes, washed in running tap water for 5 minutes, and differentiated in 1% acid alcohol for 30 seconds. After bluing in 0.2% ammonia water for 30 seconds, they were washed in running tap water for 5 minutes and rinsed in 95% alcohol. Then, they were counterstained in eosin-phloxine solution for 30 seconds and dehydrated through 95% alcohol, 2 changes of absolute alcohol, 5 minutes each. They were cleared in 2 changes of xylene, 5 minutes each and mounted with xylene based mounting medium.

### Immunohistochemical staining

Antigen retrieval was done in microwave (Bosch®, 700 watt) for 3min x90o C. They were subjected to a heating process in a microwave oven at 700 watts in a citrate buffer (pH 6) solution for proteolysis. Sections were washed in 3×5 min PBS and incubated with hydrogen peroxide (K-40677109, Germany, MERCK) for 15 min. Sections were washed in 3×5 min PBS min and blocked with Ultra V Block (lot # PHL150128, Thermo Fischer, USA) for 8 min. After draining, primary antibodies were directly applied to sections distinctly Osteonectin monoclonal antibody (SPARC), (cat # 33-5500,1:100, Thermo Fischer, USA) and Osteopontin MA5-17180, 1:100, Thermo Fischer, USA). Sections were incubated and left overnight at 4°C. Sections were washed in 3×5 min PBS and then incubated with Biotinylated Secondary Antibody (lot # PHL150128, Thermo Fischer, USA) for 20 min. After washing with PBS, Streptavidin Peroxidase (lot # PHL150128, Thermo Fischer, USA) was applied to sections for 15 min. Sections were washed in 3×5 min PBS and DAB (lot # HD36221, Thermo Fischer, USA) were applied to sections up to 10 min. Slides showing reaction were stopped in PBS. Counter staining was done with Harris's Haematoxylin for 45 sec, dehydrated through ascending alcohol and cleared in xylene. Slides were mounted with Entellan and examined under light microscope (Zeiss, Germany).

### Semi-quantitative scoring of histopathologic parameters

A semi-quantitative scoring was determined by examining osteoblast, osteocyte, and osteoclast cells in the bone tissue. During the histological sections obtained after routine histological follow-up, 15 different areas were scanned for each slide and the mean value of the 10 randomly selected cells was obtained. As a result of these averages, 10 mean scores were obtained for each group of animals and these data were analyzed statistically. Decimals were converted to integers while averages were obtained before statistical analysis. Similar semi-quantitative methods have been used in previous histochemical studies of bone tissue^21^ ^–^ ^23^.

### Statistical analysis

Statistical analyses were performed with SPSS 24.0 for Windows. Data of parameters were evaluated with Non parametric Kruskal Wallis test and multiple comparison was evaluated with Post Hoc Dunnet T3 test. Results of scores were given in Table 1 as mean rank and mean±standard deviation (SD). Mean±SD values were obtained as a result of descriptive Post Hoc test and the results were considered statistically significant for p<0.05.

**Table 1:** Statistical results of groups. Different superscript on p value column shows significantly difference between groups (*; p=0.00, **; p<0.01).

Parameter	Groups	n	Mean±SD	Mean Rank	p value
	(1) Control (Defect)	10	0,80±0.63	5.90	*(2),*(3)
Osteoblast cells	(2) Defect+Graft	10	2.30±0.48	16.55	*(1),**(3)
	(3) Defect+Graft+RSV	10	3.30±0.67	24.05	*(1),**(2)
	(1) Control (Defect)	10	0.70±0.67	6.55	**(2),*(3)
Osteocyte cells	(2) Defect+Graft	10	2.10±0.73	16.10	**(1),**(3)
	(3) Defect+Graft+ RSV	10	3.30±0.82	23.85	*(1),**(2)
	(1) Control (Defect)	10	3.60±0.51	25.10	*(2),*(3)
Osteoclast cells	(2) Defect+Graft	10	2.10±0.56	15.45	*(1),*(3)
	(3) Defect+Graft+ RSV	10	0.90±0.51	5.95	*(1),*(2)

## Results

### Histopathological scoring results

**Figure 1:**
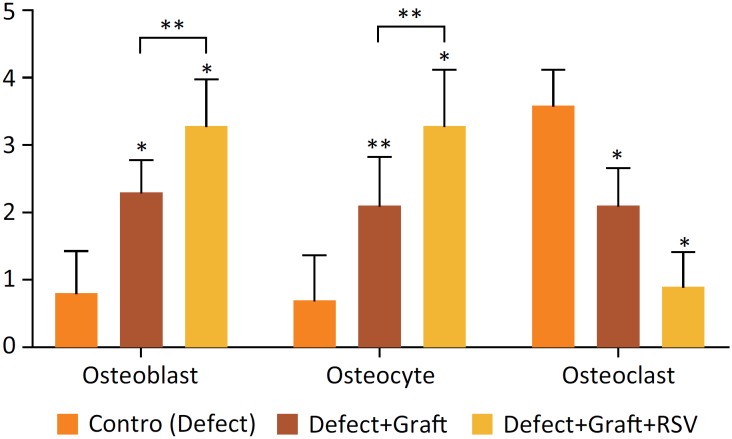
Graphic showing statical results of histopathological activity scores of osteoblast, osteocyte and osteoclast cells. Different superscript in each graph bar shows significantly difference between groups (*; p=0.00, **;p<0.01).

The histopathological results of the present study were evaluated under light microscope. Histopathological findings of control and other experimental groups were compared (Table 1, and Fig. 1).

Osteoblast and osteocyte cell activity was weak in the defect group. But in experimental groups, osteoblast and osteocyte activity was statistically more prominent as shown in Table 1 and Figure 1. However, osteoclast cells increased in the defect group, while the experimental groups decreased in Table 1 and Figure 1. Significant findings were statistically found between the groups.

### Histopathological evaluation

**Figure 2:**
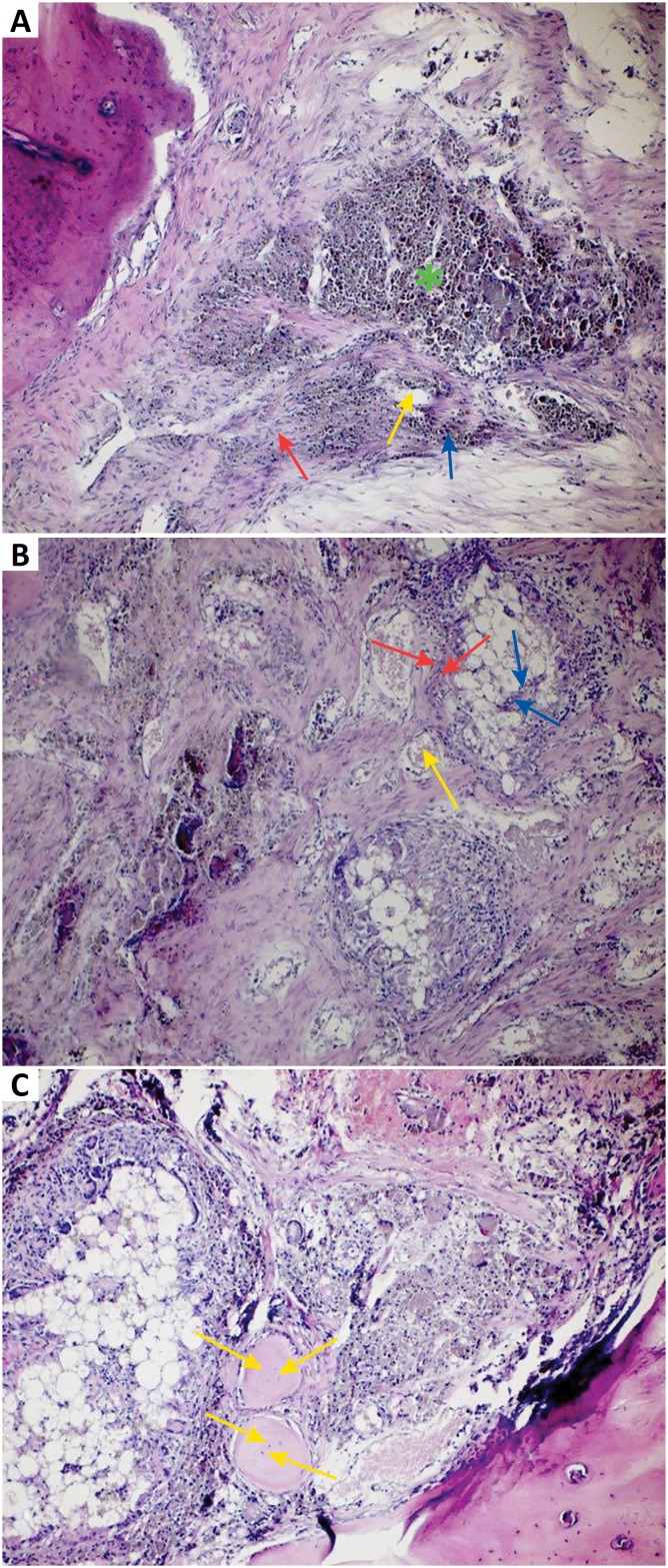
**A.** Hematoxylin and eosin staining, original magnification x20 (Control group). Intensive infiltration *(green star),* congestion of blood vessels *(yellow arrow),* increase in osteoclasts and necrotic changes degeneration in osteoblast cells *(blue arrow)* and pyknotic nucleus in osteocyte in the defect area *(red arrow).* **B.** Hematoxylin and eosin staining, original magnification x20 (Defect + graft group). Reduction of inflammation, congestion of blood vessels (*yellow arrow*), an increase in osteblastic activity *(red arrows),* progression of osteocyte development in graft area *(blue arrows).* **C.** Hematoxylin and eosin staining, original magnification x40 (Defects + graft + RSV group). An increase in osteoinductive effect, formation of bone matrix of osteoblasts and new bone formation (*yellow arrows*).

1. **Control (defect) group:** Intensive infiltration, congestion of blood vessels, increase in osteoclasts and necrotic changes were observed in the defect area. Degeneration of osteoblast cells, pyknotic changes in osteocyte cell nuclei and degenerative changes in collagen fibers were also observed (Fig. 2a).
2. **Defect + graft group:** Reduction of inflammation, congestion of blood vessels, increased osteblastic activity, osteoinductive effect, progression of osteocyte development and increased collagen fibers in connective tissue were seen (Fig. 2b).
3. **Defect + graft + RSV group:** It was observed that osteoblasts started to secrete bone matrix and accelerated osteoinductive effect with increased osteopregenitor activity. The development of mature bone structure was apparent with expanding new bone trabeculae (Fig. 2c).

### Immunohistochemical examinations

**Figure 3:**
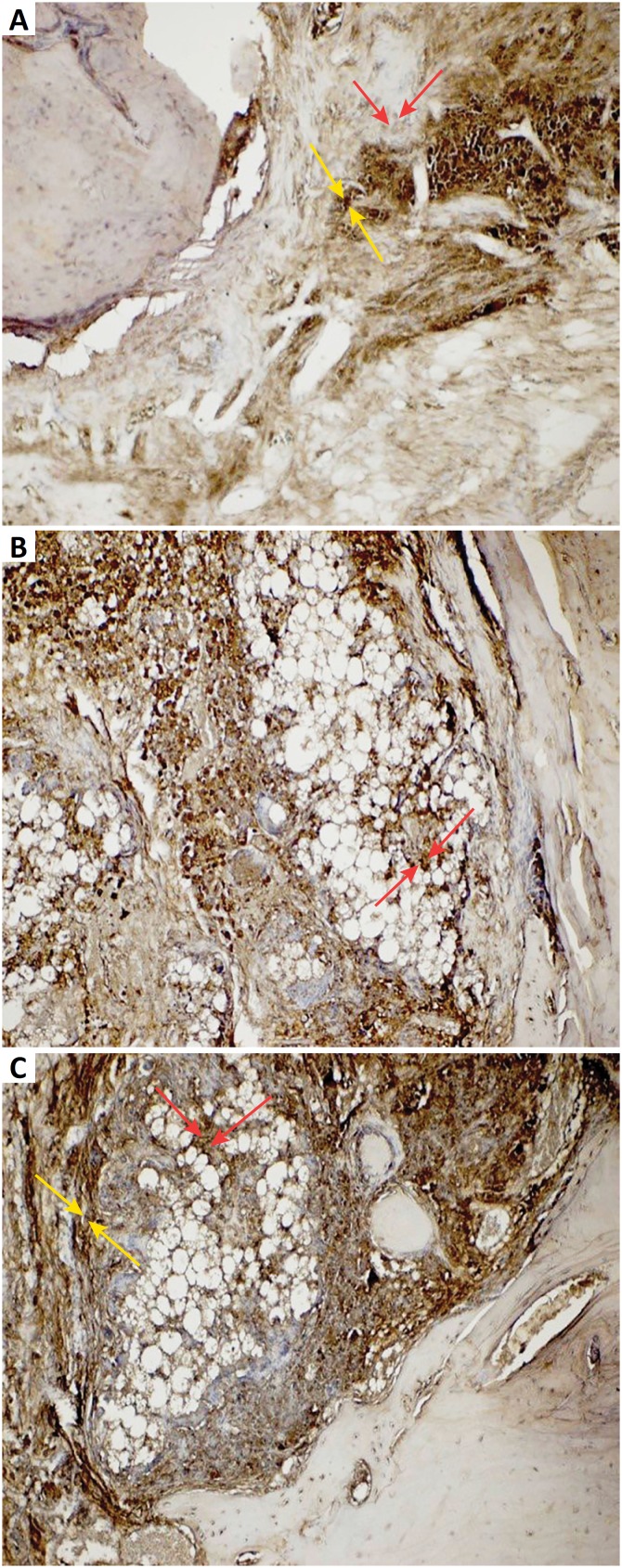
**A**. Osteopontin immunostaining, original magnification x40 (Control group). Increase of osteopontin expression in osteoclast cells *(yellow arrows),* negative osteopontin expression in osteoblast cells *(red arrows).* **B.** Osteopontin immunostaining, original magnification x40 (Defect + graft group). Positive osteopontin expression of osteblast cells in small bone trabeculae *(red arrows).* **C.** Osteopontin immunostaining, original magnification x40 (Defects + graft + RSV group). Positive osteopontin expression of osteoblasts *(yellow arrows),* and osteocytes in new bone trabeculae (*red arrows*).

**Figure 4:**
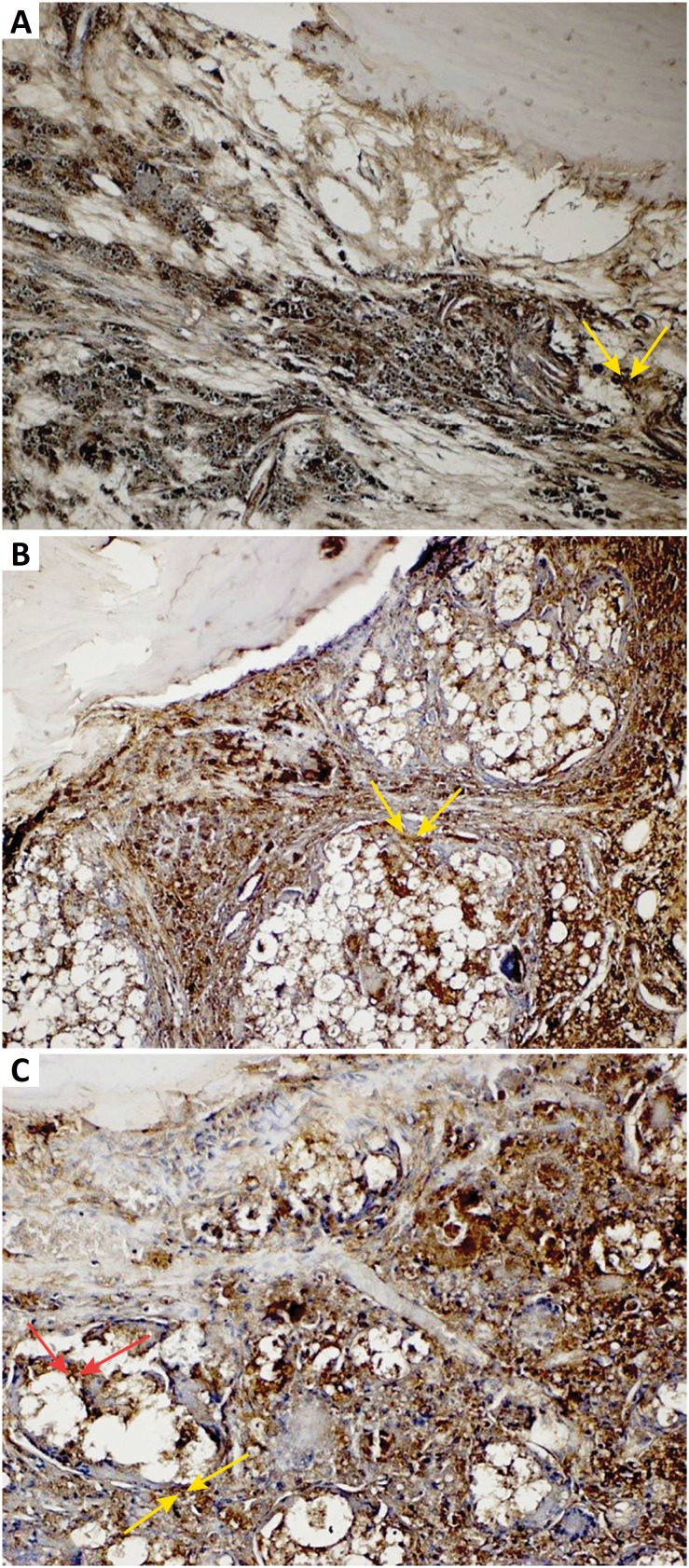
**A**. Osteonectin immunostaining, original magnification x40 (Control group). Positive osteonectin expression in inflammatory cells and osteoclastic cells around the blood vessels within the defect area *(yellow arrows).* **B.** Osteonectin immunostaining, original magnification x40 (Defect + graft group). Positive osteonectin expression in the osteblasts around the small bone trabeculum in the graft area *(yellow arrows).* **C.** Osteonectin immunostaining, original magnification x40 (Defects + graft + RSV group). Positive osteonectin expression of osteoblast (yellow arrows), and osteocyte cells in new bone trabeculae (*red arrows*).

1. **Control (defect) group:** An increase in osteopontin expression was observed in osteoclast cells and inflammatory cells around the fractured bone trabeculae and blood vessels. Also, negative osteopontin expression was observed in osteoblast cells (Figure 3a). Positive osteonectin expression was observed in inflammatory cells, collagen fibers and osteoclastic cells around the blood vessels within the defect area (Fig. 4a).
2. **Defect + graft group:** In addition to the graft area, positive osteopontin expression was observed in clustered inflammatory cells and osteoclast cells. Positive osteopontin expression was observed in osteoblast cells in small bone trabeculae with an increase in osteblastic activity in the graft (Fig. 3b). Osteonectin expression in the osteblasts around the small bone trabeculum was increased in the graft area. Osteonectin expression was positive in inflammatory cell and osteclast cells in defect area (Fig. 4b).
3. **Defect + graft + resveratrol group:** Bone trabeculae outside the graft area seems to have started to develop into the enlarged mature bone. Osteopontin positive expression was observed in osteoblast and osteocyte cells in this bone trabeculae. Graft area was reduced and positive osteopontin expression was observed in osteoblasts and osteocytes cells in bone trabeculae (Fig. 3c). It was extended from the calvarial bone to the graft area with contraction in the graft area. New bone trabeculae was formed and increased osteonectin expression in osteoblast and osteocyte cells (Fig. 4c).

## Discussion

Reconstructive surgery is important in large bone defects for maintaining rapid improvement. Rat calvarial defect has been described to evaluate bone regeneration and can be used to screen different biomaterials or tissue engineering structures before switching to larger animals for potential translation of the craniofacial complex into human applications^6^. The improvement in bone damage depends on the defect morphology and graft material. In this context, researches based on experimental calvarial defect models are performed to find out materials with the potential of having osteopromotive effect in bone repair or regeneration^24^.

Different bone graft materials have been used for bone regeneration, closure of osteotomy openings, and alveolar augmentation in oral and maxillofacial surgeons^25^. Natural coral-derived grafts and synthetic bone graft materials are used in alveolar crest elevation, intra-bone defects, material loss fractures, facial bone defects, orthognathic surgery, and maxillary sinus ground^26^ ^–^ ^28^. Alternatively, allografts are being used, which are bone tissues of the same species as the recipient but derived from genetically distinct individuals. It can be used as fresh frozen bone, frozen dried bone and demineralized bone matrix. Mah *et al*.^29^ stated that the allograft material could not be increased beyond the control levels of bone formation, but instead the advantage of the implant materials could be included in the healing zone of the material and in filling the defect by rapid bridging of the wound.

Resveratrol has been reported to be a potential drug for low-grade inflammatory diseases, which can subsequently prevent inflammation-induced bone loss and therefore lead to bone distortion. However, the results described that RSV is a more general bone-protecting agent that affects osteoblasts independently of inflammation^12^. In a study on steroid-induced osteonecrosis of femoral head, RSV application has improved local blood flow to the bone in the rabbit model. Due to its anti-inflammatory effects, RSV protects the vascular endothelium and reduces tissue hypoxia and thrombosis^30^. Casati *et al*.^31^ reported that RSV reduced alveolar bone loss in periodontal disease in rats, modulated local cytokine levels and had anti-inflammatory potential. In our study, rapid development of new bone trabeculae on the 28^th^ day of the defect region was observed and also an increase in osteoblastic and osteoclastic activities was accelerated by graft material and RSV application. Local RSV administration has been shown to have positive effects on the formation of new bone by stimulating bone repair in the intermaxillary suture and shortening the time required for postoperative retention in rats exposed to surgical maxillary enlargement^32^. In the study, two calvarial defects were created and one screw-shaped titanium implant was inserted in the tibia of rats that were subjected to 10 mg/kg/die RSV administration by gavage for 30 days. The histomorphometric analysis resulted in a reduced remaining defect in the RSV group. According to their resuls, RSV administration improves the repairing of critical bone defects and biomechanical retention of titanium implants^33^. Durbin *et al*.^34^ exhibited effects of RSV supplementation on disuse and age-related bone loss and noted increased osteoblast bone formation due to reduced inflammation. They concluded that RSV supplementation appeared to provide a feasible dietary therapy for preserving the skeletal system. However, a previous study conducted by the same study group on providing RSV in the low supplementation range for a short duration that whether it prevents bone loss during mechanical unloading in the tibia of hindlimb-suspended (HLS) rats indicated that RSV supplementation of 6-month-old HLS male rats had no bone protective effects and possibly even detrimental bone effects^35^. RSV was also studied in type 2 diabetic patients as they have increased fracture risk by Bo *et al*.^36^ and they verified that RSV prevented bone density loss. Pino *et al*.^37^ examined the effect of RSV on critical-sized calvarial defects of diabetic rats via histometric and gene expression analysis. They found out positively influenced bone repair in animals with induced DM.

In the literature, the doses of resveratrol administered have been reported to be given orally between different doses per day in animal models. It has been shown that a dose of 5 mg/kg resveratrol can prevent ovariectomy-induced decreases in femoral bone strength^38^. Lee *et al*.^39^ suggested that RSV at 10 mg/kg showed not to affect bone health in normal rats, but to aggravate the bone damage in methotrexate-treated rats. On the other hand, they also supported that RSV supplementation at low dosage (1 mg/kg) preserved the growth plate, primary spongiosa, bone volume, and lowered the adipocyte density of tibia bone. In calvarial defect models, they proposed that nondiabetic animals presented with the highest levels of osteopontin and Runx2 mRNA compared with all other groups at dosage of 10 mg/kg^37^, and resveratrol significantly affected the levels of expression of bone morphogenetic protein 2, bone morphogenetic protein 7, and osteopontin^33^. In our study, although the dosage of resveratrol was at 5 mg/kg/day, osteopontin expression was increased in osteoblasts and osteocytes cells of new bone trabeculae. On the other hand, in another study on femur healing histomorphometrically and to evaluate the effects of resveratrol on negative effects of cigarette smoke showed that areas of new bone formation in the resveratrol and control groups were higher than in the smoking and smoking+resveratrol groups of rats that were given 20 mg/kg body weight resveratrol^40^. Apart from these, the effects of resveratrol cell culture models in relation to bone tissue were investigated in different studies^41^ ^–^ ^43^.

Osteopontin (OPN) is a noncollagenous matrix protein and known to play a role in cell adhesion, migration, survival, and bone remodeling^44^ ^,^ ^45^. OPN is produced abundantly by osteoblasts and osteocytes as a phosphorylated, secreted extracellular matrix protein^46^ ^,^ ^47^. OPN is eagerly bound to mineral crystal surfaces to inhibit growth when the mineralizing bone matrix is loaded into the stack phase. OPN facilitates osteoclast binding and directs mineral deposition by affecting crystal shape and size^48^. It has been reported to increase the effect of paracrine cytokines produced by stromal/ osteoblastic cells, thus promoting the proliferation or differentiation of hematopoietic precursors^49^. In our study, OPN expression showed positive reaction in osteoblasts in small bone trabeculae after graft application (Fig. 3b).

Osteonectin is secreted by osteoblasts in bone during bone development and remodeling. Osteonectin helps to connect bone mineral and collagen fibrils to each other^50^. The osteonectin protein was localized in mineralized bone trabeculae and was found to be at a higher level in the matrix than the bone cells. Meanwhile, estrogens increase the differentiation of osteoblast cells and stimulate bone matrix mineralization, arrange the expression of non-collagenous proteins such as type I collagen and osteopontin, osteocalcin, osteonectin and the like^51^. A study of Lacin *et al*.^52^ reported the effects of allopurinol treatment in rat calvaria defects may induce osteoblastic activity, matrix development, mature bone cell formation and new bone formation when used with alloplastic graft. In this study, it was observed that osteoinductive effect has been increased as RSV causes the increment of collagen activity in connective tissue by decreasing callus development of allograft material. The inductive effect of osteopregenitor cells has led to the development of invasive osteoblast cells, leading to the development of matrix and osteoblast cells in new bone formation.

To summarize, the present study demostrated that allograft application induced bone formation and was suitable as a material in the rat calvarial defect. After 28 days of RSV treatment along with the application of graft material, osteoclastic activity was slowed down; matrix formation and maturation of osteocyte cells with the increase of osteoplastic activity has shaped new bone trabeculae.

## Conclusion

Resveratrol treatment was thought to be an alternative and supportive drug for implant application by inducing new bone formation in the calvaral defect region as a result of short-term treatment. Further studies with different application routes, dosages and time intervals are needed to widely understand its action.
